# Coverage and factors associated with mother and newborn skin-to-skin contact in Nigeria: a multilevel analysis

**DOI:** 10.1186/s12884-021-04079-8

**Published:** 2021-09-04

**Authors:** Michael Ekholuenetale, Amadou Barrow, Faith Owunari Benebo, Ashibudike Francis Idebolo

**Affiliations:** 1 Department of Epidemiology and Medical Statistics, Faculty of Public Health, College of Medicine, University of Ibadan, Ibadan, Nigeria; 2 Department of Public & Environmental Health, School of Medicine & Allied Health Sciences, University of The Gambia, Kanifing, The Gambia; 3 Clinical Case Management Unit, Management Sciences for Health, Abuja, Nigeria; 4 Department of International Public Health, Liverpool School of Tropical Medicine, Liverpool, UK

**Keywords:** Kangaroo mother care, Postpartum, Neonatal, SSC, Nigeria, Inequality

## Abstract

**Background:**

Mother and newborn skin-to-skin contact (SSC) is an immediate postpartum intervention known to improve the health of newborn and mothers alike. Albeit, there is paucity of data that explored the coverage or factors associated with SSC in Nigeria. Therefore, we aimed to explore the coverage and hierarchical nature of the factors associated with SSC among women of reproductive age in Nigeria.

**Methods:**

The 2018 Nigeria Demographic and Health Survey (NDHS) data was used for this study. Data on 29,992 women who had ever given birth were extracted for analysis. SSC was the outcome variable as determined by women’s report. A multivariable multilevel logistic regression model was used to estimate the fixed and random effects of the factors associated with SSC. Statistical significance was determined at *p*< 0.05.

**Results:**

The coverage of SSC was approximately 12.0%. Educated women had higher odds of SSC, when compared with women with no formal education. Those who delivered through caesarean section (CS) had 88% reduction in SSC, when compared with women who had vaginal delivery (OR= 0.12; 95%CI: 0.07, 0.22). Women who delivered at health facility were 15.58 times as likely to practice SSC, when compared with those who delivered at home (OR= 15.58; 95%CI: 10.64, 22.82). Adequate ANC visits and low birth weight significantly increased the odds of SSC. Women from richest household were 1.70 times as likely to practice SSC, when compared with women from poorest household (OR= 1.70; 95%CI: 1.04, 2.79). There was 65% reduction in SSC among women with high rate of community non-use of media, when compared with women from low rate of community non-use of media (OR= 0.35; 95%CI: 0.20, 0.61).

**Conclusion:**

SSC coverage was low in Nigeria. Moreover, individual, household and community level factors were associated with SSC. More enlightenment should be created among women to bring to limelight the importance of SSC specifically to newborn’s health.

## Introduction

Globally, about 2.5 million newborn die in their first month of life, accounting for 47% of under-five death [1]. Although, there has been a 50% reduction in the number of neonatal death from 5 million in 1990 to 2.5 million in 2018, still, the decrease in neonatal death has been slow, when compared to the overall reduction in post-neonatal death among the under-five [1]. Several causes have been attributed to childhood death [2]. SSC is a recommended natural intervention for improving neonatal survival rates. It is a component of thermal care, in the light of drying, wrapping and delayed bathing. The World Health Organization (WHO) recommends that after delivery, every newborn should receive immediate newborn care, which includes thermal care, hygienic cord care and early initiation of breastfeeding [3]. Therefore, physiological stability, thermoregulation, blood glucose, early breastfeeding initiation could be higher in newborns exposed to SSC. Moreover, SSC is beneficial to mothers with postpartum depressive symptoms as it reduces maternal anxiety and enhances bonding and caregiving behaviour towards the newborn [4]. In prematurity, SSC can improve survival by providing warmth, love, stimulation and protection from nosocomial sepsis. In resource-poor settings, this can be life-saving as there is often lack of expertise or technology to effectively manage complications [5].

Similarly, SSC has been known as a safe substitute to reduce neonatal mortality in resource-poor settings [6]. However, despite the overwhelming evidence that supports the benefits of SSC, its uptake and adoption continues to be low [7]. Numerous factors have militated against improved practice of SSC. For instance, healthcare workforce shortages, closed-mindedness, safety concerns, interruptions from other healthcare practitioners on medical procedures for infant or mother are some of the notable barriers to practice SSC [8]. Conversely, some enabling factors to SSC include improved healthcare providers’ knowledge on the benefits of SSC, positive experiences of the effect of SSC on the mother and newborn and women’s acceptance or willingness which are commonly influenced by cultural, educational, religious background amongst others [8].

The health care system of any setting has a role to play on the implementation of practices recommended for positive health outcomes. Unfortunately, Nigeria has a weak health care delivery system that contributes to adverse maternal and child health outcomes [9]. Promoting institutional delivery could in turn increase the practice of SSC due to the fact that skilled birth attendants could have the knowledge of SSC. A major limitation to that assertion is the health delivery system in Nigeria is inadequately funded [10], to provide proper training and on-the-job training of health workers in the delivery of modern practices including maternal health care delivery. No doubt, this has largely favoured home deliveries with traditional birth attendants. This is evident in the average national indices of maternal and child health care, particularly under-utilisation of vital maternal health care services such as skilled care delivery which is among the poorest in sub-Saharan Africa [11]. The Integrated Maternal, Newborn and Child Health (IMNCH) Strategy currently being implemented in the country seeks to boost utilisation of essential maternal and child health services across the continuum of care [11]. Nevertheless, the coverage of the IMNCH interventions is remains low particularly in hard-to-reach areas of the country [12].

With efforts to meet the targets of the Sustainable Development Goals (SDGs) by 2030, to reduce neonatal mortality to as low as 12 per 1000 live births [13], it is crucial to incorporate all proven measures aimed at reducing neonatal mortality and improving newborn’s health into health care programmes. Hence, to effectively scale-up SSC as an intervention to reduce neonatal mortality in Nigeria, it is necessary to understand the determinants of practise or non-practise of SSC [14]. In this novel study, we aimed to explore the practice and factors associated with SSC in Nigeria using the most recent NDHS data.

## Methods

### Data source

In this analysis, we used cross-sectional, nationally representative data. For study, NDHS 2018 data was collected from 29,992 women who had ever given birth. A three-stage sampling stratification was involved in the sampling design, in which respondents were first stratified by urban versus rural dwellings, then randomly selected enumeration areas (EAs) within each stratum. Finally, using equal likelihood sampling, households within each EA were then chosen for the survey. In the calculation of survey weights, this three-stage sampling procedure was taken into account and applied to ensure the representativeness of the sample with respect to the general population. Data for this analysis is obtained from the questionnaire of individual women. The data is available in the public domain and accessed at; http://dhsprogram.com/data/available-datasets.cfm. Details of DHS sampling procedure has been reported previously [15].

### Variables of the study

#### Outcome

Mother and newborn skin-to-skin contact was derived from the question; “*Was child put on mother's chest and bare skin after birth*”. This was measured dichotomously and coded as “1” if a woman answered “yes” and “0” if otherwise.

### Individual-level factors

- Maternal age: 15-19, 20-24, 25-29, 30-34, 35-39, 40-44, 45-49.
- Residential mobility: internal immigrant (if a respondent has lived less than 5 years in the current location) vs. indigenous (if a respondent has lived up to 5 years and above in the current location).
- Educational achievement: no formal schooling, primary and secondary or higher education.
- Religion: Christianity, Islam and the Traditions of Africa.
- Media exposure: was measured dichotomously (yes vs. no) whether a respondent used any newspaper/magazine, radio, TV or internet; Number of children ever born: 1-2, 3-4 and over 4 children.
- Child wanted while pregnant: then, later and no longer wanted.
- Health insurance: protected vs. not covered.
- Job status: working vs. not working.
- Childbirth mode: caesarean vs. vaginal.
- Location of delivery: health facility vs. home.
- ANC visits: no visit, 1-3, 4 and above.
- Type of birth: singleton vs. multiple.
- Sex of child: male vs. female.
- Preceding birth interval: <2years, 2-3years, >3-4years, >4years, firstborn.
- Birth weight: low birth weight (<2.5kg) vs. average (≥2.5kg). These factors were included based on previous studies which examined the factors associated with SSC [16–19].

### Household-level factors

The size of the household was based on the total number of individuals residing together; 1-4, 5-8 and over 8 individuals. Household wealth quintile: To assign the wealth indicator weights, Principal Components Analysis (PCA) was used. This protocol assigned scores and standardized variables for the wealth predictor, such as; Main wall material, main roof material, sanitation facilities, water supply, radio, television, electricity, refrigerator, cooking fuel, furniture, number of people per room, bicycle, motorcycle/scooter, car/truck, main floor material, main wall material. Scores of the factor coefficient (factor loadings) and z-scores were determined. The indicator values have been multiplied by the loadings for each household and summed to produce the household’s wealth index value. The standardized z-score was used to disentangle the overall assigned scores to poorest, poorer, middle, richer and richest class [20, 21].

### Community-level factors

Residential status: urban vs. rural. Geographical area: North Central, North East, South East, South South and South West, North East, North West. Non-exposure media concentration in the group (whether or not more than 50 percent of the cluster population does not read newspapers/magazines, do not listen to radio, watch TV or do not use the internet). Uneducated women's concentration in the community (whether or not more than 50 percent pop of the cluster had no formal education). Poverty concentration in the society (whether or not more than 50 percent of the population are in the least wealth quintiles). This approach is similar to the methods of a previous study [22].

### Ethical consideration

In this analysis, with all identifier information removed, we used population-based secondary datasets available in the public/online domain. Access was given to the authors by MEASURE DHS/ICF International to use the data. The DHS complies with requirements to ensure the security of the privacy of respondents. ICF International makes sure the survey is consistent with the U.S. Department of Health and Human Services regulations for the respect of human subjects. No further approval was required for this study. More details about data and ethical standards are available at http://goo.gl/ny8T6X.

### Statistical analysis

For data strata, cluster and sample weights, we used the 'svy' module to modify. To estimate the fixed and random effects of the factors associated with SSC, a multivariable multilevel logistic regression model was used. For binary answer, we defined a 3-level model. A single woman (at level 1), a family (at level 2) living in a group (at level 3). We had five models built. To break down the amount of variance between group and household levels, the first model, an empty or unconditional model without any explanatory variables, was defined. In order to understand the variations of the community and households, the null or empty model is important and we used it as a guide to estimate how many household and community variables were able to explain the observed variation. Furthermore, we used it to explain the use of the multilevel statistical method, since it recommended the use of single-level logistic regression if the population variance was not important in the empty model. Statistical significance was determined at *p*< 0.05. Data analysis was conducted using Stata Version 14 (StataCorp., College Station, TX, USA).

### Fixed and random effects

The results of fixed effects (association measures) with a 95 percent confidence interval were reported as adjusted odds ratios (AORs) (CI). The Intra-class Correlation (ICC) and Median Odds Ratio (MOR) [23] evaluated the possible contextual effects. We calculated the resemblance between respondents using ICC in the same household and within the same group. The ICC reflects the proportion of the total variance in the likelihood of SSC that is connected to the level of the household and society. The MOR calculates the variance of the second or third stage (household or community) as an odds ratio and estimates the likelihood of SSC that can be related to the background of the household and community. Unity MOR does not mean any variance in the household or community variance. The higher the MOR, the more significant the contextual effects for understanding the likelihood of SSC are, on the other hand. The ICC was determined according to the formula used by Snijders and Bosker [24] by the linear threshold, while the MOR is a measure of unknown heterogeneity of clusters. The Bayesian and Akaike Knowledge Criterion is used to assess how well our various models match. A lower value implies a better fit of the model on the Akaike or Bayesian Information Criterion [25].

## Results

### Background characteristics

Table 1 showed the coverage of SSC was approximately 12.0% among Nigerian women. Based on the results, women with secondary and higher education had SSC coverage of 14.6% and 19.7% respectively. Those of African Tradition Religion (ATR) had 48.9% of SSC coverage. SSC coverage among women who had exposure to media (14.3%), health insurance coverage (17.6%), vaginal delivery (12.2%), delivery at health facility (19.0%), 4+ ANC visits (14.7%), low birthweight (23.0%), living in the richest household (17.2%), urban residence (14.3%), South South geographical region (19.9%), low community non-use of media or poverty (14.3%) and low community illiteracy (15.5%) were also presented respectively.

**Table 1:** Coverage of SSC across women’s characteristics in Nigeria

Variable	Number of women (%)	Mother and newborn skin-to-skin contact		p
		Yes (12.0%)	No (88.0%)	
**Age**				0.015*
15-19	1197 (4.0)	10.9	89.1	
20-24	4320 (14.4)	12.4	87.6	
25-29	6107 (20.4)	12.0	88.0	
30-34	5536 (18.5)	12.4	87.6	
35-39	5155 (17.2)	11.9	88.1	
40-44	3905 (13.0)	12.2	87.8	
45-49	3772 (12.6)	8.9	91.1	
**Residential mobility**				0.562
Internal immigrant (<5years)	4414 (14.7)	12.3	87.7	
Native (5+years)	25578 (85.3)	11.9	88.1	
**Educational attainment**				<0.001*
No formal education	12455 (41.5)	8.7	91.3	
Primary	5351 (17.8)	11.7	88.3	
Secondary	9549 (31.8)	14.6	85.4	
Higher	2637 (8.8)	19.7	80.3	
**Religion**				<0.001*
Christianity	13808 (46.0)	12.4	87.6	
Islam	15909 (53.0)	11.2	88.8	
ATR/others	275 (0.9)	48.9	51.1	
**Media exposure**				<0.001*
Has no exposure	10923 (36.4)	8.4	91.6	
Has exposure	19069 (63.6)	14.3	85.7	
**Total children ever born**				<0.001*
1-2	9408 (31.4)	13.2	86.8	
3-4	8493 (28.3)	11.9	88.1	
4+	12091 (40.3)	11.0	89.0	
**Wanted child when became pregnant**				0.194
Then	19054 (87.4)	12.1	87.9	
Later	1976 (9.1)	11.3	88.7	
No more	762 (3.5)	10.4	89.6	
**Health insurance**				<0.001*
Not covered	29196 (97.4)	11.9	88.1	
Covered	796 (2.6)	17.6	82.4	
**Mode of delivery**				<0.001*
Caesarean section	647 (3.0)	7.3	92.7	
Vaginal	21055 (97.0)	12.2	87.8	
**Place of delivery**				<0.001*
Health facility	9351 (42.9)	19.0	81.0	
Home	12441 (57.1)	6.8	93.2	
**ANC visits**				<0.001*
No visit	5365 (25.0)	5.8	94.2	
1-3 visits	3793 (17.7)	10.8	89.2	
4+ visits	12307 (57.3)	14.7	85.3	
**Child sex**				0.186
Male	15345 (51.2)	12.3	87.7	
Female	14647 (48.8)	11.7	88.3	
**Preceding birth interval**				<0.001*
<2years	5196 (17.3)	12.4	87.6	
2-3 years	9748 (32.5)	10.8	89.2	
>3-4years	5150 (17.2)	11.5	88.5	
>4years	5186 (17.3)	13.3	86.7	
First born	4712 (15.7)	13.4	86.6	
**Birthweight**				<0.001*
Low birthweight (<2.5kg)	379 (1.7)	23.0	77.0	
Normal (≥2.5kg)	21413 (98.3)	11.8	88.2	
**Household wealth quintile**				<0.001*
Poorest	6296 (21.0)	7.8	92.2	
Poorer	6418 (21.4)	10.5	89.5	
Middle	6350 (21.2)	12.4	87.6	
Richer	5946 (19.8)	14.4	85.6	
Richest	4982 (16.6)	17.2	82.8	
**Household size**				0.001*
1-4	9719 (32.4)	13.2	86.8	
5-8	13444 (44.8)	11.9	88.1	
8+	6829 (22.8)	10.9	89.1	
**Residential status**				<0.001*
Urban	11259 (37.5)	14.3	85.7	
Rural	18733 (62.5)	10.8	89.2	
**Geographical region**				<0.001*
North Central	5452 (18.2)	8.3	91.7	
North East	5694 (19.0)	14.6	85.4	
North West	7745 (25.8)	11.5	88.5	
South East	3617 (12.1)	5.9	94.1	
South South	3501 (11.7)	19.9	80.1	
South West	3983 (13.3)	13.4	86.6	
**Community non-use of media**				<0.001*
Low	20082 (67.0)	14.3	85.7	
High	9910 (33.0)	8.1	91.9	
**Community poverty**				<0.001*
Low	17829 (59.4)	14.3	85.7	
High	12163 (40.6)	9.1	90.9	
**Community illiteracy**				<0.001*
Low	13878 (46.3)	15.5	84.5	
High	16114 (53.7)	9.5	90.5	

*Significant at *p*<0.05

### Measures of associations

Results from Table 2 showed various models used to examine the hierarchical nature of the factors associated with SSC. In Model II, we examined the individual level factors. Women aged 45-49 years had 53% significant reduction in SSC, when compared with those aged 15-19 years. Those with secondary (OR= 1.33; 95%CI: 1.00, 1.76) and tertiary education (OR= 2.50; 95%CI: 1.68, 3.72) were more likely to have SSC, when compared with women with no formal education. In addition, women who delivered at health facility were 15.59 times as likely to practice SSC, when compared to those who delivered at home (OR= 15.59; 95%CI: 10.41, 23.36). ANC visits and low birth weight significantly increased SSC among Nigerian women. Results from Model III showed that women from higher household wealth quintiles were more likely to practice SSC, when compared with the poorest women. Conversely, women from large household size had significant reduction in SSC, compared with women from small household size. The community level analysis (Model IV) showed that geographical region had significant association with SSC. Furthermore, high level of community non-use of media and illiteracy had 75% (OR= 0.25; 95%CI: 0.15, 0.41) and 52% (OR= 0.48; 95%CI: 0.30, 0.78) significant reduction in SSC, when compared with low level of community non-use of media and illiteracy respectively.

**Table 2:** Fixed effect of individual-, household- and community-level factors associated with SSC in Nigeria

Variable	Model I	Model II	Model III	Model IV	Model V
**Individual level factors**					
**Age group of women**					
15-19		1.00			1.00
20-24		0.99 (0.64-1.51)			0.99 (0.66-1.50)
25-29		0.93 (0.59-1.47)			0.98 (0.63-1.51)
30-34		0.93 (0.57-1.51)			0.99 (0.62-1.60)
35-39		0.81 (0.48-1.36)			0.90 (0.54-1.49)
40-44		0.93 (0.52-1.64)			1.02 (0.59-1.79)
45-49		0.47 (0.23-0.96)*			0.53 (0.27-1.06)
**Educational attainment**					
No formal education		1.00			1.00
Primary		0.98 (0.74-1.30)			1.08 (0.82-1.42)
Secondary		1.33 (1.00-1.76)*			1.41 (1.06-1.86)*
Higher		2.50 (1.68-3.72)*			2.35 (1.57-3.50)*
**Religion**					
Christianity		1.00			1.00
Islam		1.83 (1.35-2.49)*			1.11 (0.80-1.54)
ATR/others		72.08 (24.68-210-57)*			55.31 (20.18-151.63)*
**Media exposure**					
Has no exposure		1.00			1.00
Has exposure		0.93 (0.75-1.16)			0.92 (0.74-1.15)
**Total children ever born**					
1-2		1.00			1.00
3-4		0.98 (0.76-1.28)			0.99 (0.75-1.30)
4+		1.13 (0.82-1.55)			1.10 (0.79-1.52)
**Health insurance**					
Not covered		1.00			1.00
Covered		1.31 (0.75-2.30)			1.13 (0.66-1.93)
**Mode of delivery**					
Caesarean section		0.12 (0.06-0.21)*			0.12 (0.07-0.22)*
Vaginal		1.00			1.00
**Place of delivery**					
Health facility		15.59 (10.41-23.36)*			15.58 (10.64-22.82)*
Home		1.00			1.00
**ANC visits**					
No visit		1.00			1.00
1-3 visits		1.54 (1.11-2.14)*			1.46 (1.06-2.01)*
4+ visits		2.48 (1.81-3.39)*			2.40 (1.78-3.24)*
**Preceding birth interval**					
<2years		1.00			1.00
2-3 years		0.82 (0.64-1.06)			0.82 (0.65-1.05)
>3-4years		0.91 (0.69-1.22)			0.90 (0.69-1.19)
>4years		1.06 (0.78-1.43)			1.01 (0.76-1.36)
First born		0.96 (0.69-1.34)			0.95 (0.69-1.32)
**Birthweight**					
Low birthweight (<2.5kg)		2.16 (1.27-3.67)*			2.10 (1.26-3.51)*
Normal (≥2.5kg)		1.00			1.00
**Household-level factors**					
**Household wealth quintile**					
Poorest			1.00		1.00
Poorer			1.49 (1.11-2.00)*		1.09 (0.79-1.50)
Middle			1.93 (1.39-2.68)*		1.14 (0.78-1.67)
Richer			3.12 (2.17-4.49)*		1.38 (0.89-2.12)
Richest			5.83 (3.84-8.83)*		1.70 (1.04-2.79)*
**Household size**					
1-4			1.00		1.00
5-8			0.83 (0.70-0.99)*		0.97 (0.77-1.21)
8+			0.74 (0.58-0.93)*		0.93 (0.70-1.24)
**Community-level factors**					
**Residential status**					
Urban				1.00	1.00
Rural				0.89 (0.60-1.31)	1.12 (0.74-1.24)
**Geographical region**					
North Central				1.00	1.00
North East				6.31 (3.48-11.44)*	11.52 (5.92-22.43)*
North West				2.52 (1.48-4.32)*	6.75 (3.62-12.56)*
South East				0.22 (0.12-0.43)*	0.18 (0.09-0.37)*
South South				2.22 (1.25-3.94)*	3.49 (1.85-6.61)*
South West				0.77 (0.43-1.36)	0.66 (0.36-1.21)
**Community non-use of media**					
Low				1.00	1.00
High				0.25 (0.15-0.41)*	0.35 (0.20-0.61)*
**Community poverty**					
Low				1.00	1.00
High				0.67 (0.41-1.09)	1.15 (0.67-2.20)
**Community illiteracy**					
Low				1.00	1.00
High				0.48 (0.30-0.78)*	1.03 (0.62-1.71)

Model I – empty null model, baseline model without any explanatory variables (unconditional model)

Model II – adjusted for only individual-level factors

Model III – adjusted for only household-level factors

Model IV – adjusted for only community-level factors

Model V – adjusted for individual-, household-, and community-level factors (full model)

*Significant at *p*<0.05

Results from the full model (Model V) showed women with secondary (OR= 1.41; 95%CI: 1.06, 1.86) and tertiary education (OR= 2.35; 95%CI: 1.57, 3.50) were more likely to have SSC, when compared with women with no formal education. Women who delivered through caesarean section had 88% reduction in SSC, when compared with women who had vaginal delivery (OR= 0.12; 95%CI: 0.07, 0.22). Furthermore, women who delivered at health facility were 15.58 times as likely to practice SSC, when compared to those who delivered at home (OR= 15.58; 95%CI: 10.64, 22.82). ANC visits and low birth weight significantly increased SSC. Women from richest household were 1.70 times as likely to have SSC, when compared with women from poorest household (OR= 1.70; 95%CI: 1.04, 2.79). Geographical region of women was significantly associated with SSC. There was 65% significant reduction in SSC among women from high level community non-use of media, when compared with with women from low level community non-use of media (OR= 0.35; 95%CI: 0.20, 0.61). See Table 2 for the details.

### Measures of variations

We presented variance and standard error (SE) for the random effects in Table 3. In Model 1 (unconditional model), there were significant variations in the odds of SSC across communities (σ^2^ = 2.68; SE= 0.21) and households (σ^2^= 2.32, SE= 0.27). The variance partition coefficient of 28.0% and 32.9% estimates for the null model implied that variance in the odds of SSC could be attributed to community and household level factors respectively. Results from the Median Odds Ratio also confirmed evidence of community and household-level factors influencing SSC. Based on estimates from the full model (Model V), it was estimated that if a women moved to another community or household with a higher probability of SSC, the median increase in their odds of SSC would be 10.88 and 8.79 fold respectively. The proportional change in variance (PCV), intra-class correlation (ICC) and model fit statistics were also estimated.

**Table 3:** Random effect estimates of individual-, household- and community-level factors associated with SSC in Nigeria

Random-effect	Model I	Model II	Model III	Model IV	Model V
**Community-level**					
Variance (SE)	2.68 (0.21)*	2.89 (0.22)*	2.59 (0.17)*	2.36 (0.15)*	2.50 (0.18)*
VPC	28.0%	27.9%	27.9%	26.5%	26.2%
PCV	Reference	-7.8%	3.4%	11.9	6.7%
MOR	12.91	15.76	11.79	9.48	10.88
ICC	45.4%	47.7%	44.9%	40.3%	42.5%
**Household-level**					
Variance (SE)	2.32 (0.27)*	2.42 (0.25)*	2.22 (0.22)*	2.22 (0.21)*	2.28 (0.23)*
VPC	32.9%	33.0%	32.8%	33.6%	33.7%
PCV	Reference	-4.3%	4.3%	4.3%	1.7%
MOR	9.13	10.11	8.32	8.34	8.79
ICC	33.9%	33.5%	33.1%	35.8%	35.2%
**Model fit statistics**					
AIC	13465.7	12157.4	13367.5	13322.9	12008.2
BIC	13489.6	12372.6	13439.4	13418.8	12343.0

Model I – empty null model, baseline model without any explanatory variables (unconditional model)

Model II – adjusted for only individual-level factors

Model III – adjusted for only household-level factors

Model IV – adjusted for only community-level factors

Model V – adjusted for individual-, household-, and community-level factors (full model)

*SE* Standard Error, *VPC* Variance Partition Coefficient, *PCV* Proportional Change in Variance, *MOR* Median Odds Ratio, *ICC* Intra-class correlation, *AIC* Akaike's Information Criterion, *BIC* Bayesian Information Criterion, *Significant at *p*<0.05

## Discussion

In this study, we explored the coverage, enablers and barriers of SSC in Nigeria, at individual, household and community levels. Based on the findings of the study, the coverage of SSC was low. Furthermore, the mode of delivery, educational attainment, place of delivery, attendance of ANC visits and LBW were significantly associated with SSC. At the household level, women belonging to the richest households were more likely to have SSC. While high rate of community non-use of media and geographical region were significantly associated with SSC at the community level.

The prevalence of SSC was found to be 12%. This is slightly higher than the report from a previous study which showed that only 10% of newborns received SSC [26]. This coverage is still very low despite widespread recommendations from WHO and evidence of proven benefits of SSC both for the mother and the newborn [18, 27, 28]. In the findings from another study using data from low, middle and high income countries, SSC coverage ranged between <1% in Tanzania to 97.8% in Croatia [27]. Nonetheless, there is dearth of empirical studies on SSC in Nigeria, but findings from a qualitative study showed that mothers did not view SSC as important for keeping the baby warm after birth as newborns were physically away from their mothers after birth [29]. Mothers may be less likely to practice SSC if they are not aware of it and/or its benefits [17, 30], or if there are prevailing cultural beliefs and practices that discourage SSC [29–31]. Health personnel factors such as lack of personnel and time constraint, lack of awareness of the practice and its benefit may also be barriers to the practice of SSC [16]. These prevailing factors could explain the reasons for low SSC in Nigeria.

An individual-level factor in this study that was strongly associated with the practice of SSC was delivery at a health facility. Women who delivered in a health facility had 15 times higher odds of having SSC immediately after delivery with their newborns. This is consistent with the findings from previous studies which showed that the practice of SSC between the mother and the newborn was significantly more common in health facility based delivery compared with home births [32, 33]. Skilled birth attendants at health facilities may be more knowledgeable about SSC than traditional birth attendants, making it more likely to be practiced in hospital deliveries [34, 35]. Furthermore, the mode of delivery was significantly associated with SSC between mothers and their newborn immediately after birth. Caesarean section decreased the odds of SSC compared with vaginal delivery. This is consistent with the findings from previous studies [36, 37]. Mothers who had caesarean section may be in pain, medicated or feel discomfort, thereby reducing the likelihood of SSC. In a previous study, hospital procedures led to the separation between mother and infant; midwives experienced obstacles to facilitate SSC due to mothers’ condition after caesarean section, lack of time and sometimes midwives felt dismissed or disappointed when they tried to communicate the benefits of SSC [38].

Furthermore, higher odds of SSC was observed among women with higher levels of education compared to mothers with no education. Educated mothers may be more knowledgeable about SSC and its benefits, they may be more likely to discuss it with their health providers and request for it even when the information is not offered by the provider. Also, mothers who had attended ANC visits had increased odds of having SSC with their newborns immediately after delivery, compared with those who had no ANC visits. Similar findings have been reported in other studies [17, 35]. ANC is an avenue to educate, counsel and prepare pregnant women for delivery. Women are educated on healthy behaviours to adopt during pregnancy and what to expect during and after delivery. At ANC visit, women can be sensitized about SSC and its benefits [35, 39]. In addition, mothers who had LBW newborns were more likely to practice SSC with their newborn immediately after birth. SSC for LBWs is a component of the WHO endorsed KMC, which also includes nutrition [40, 41]. Nigeria has recognized KMC as part of essential care for LBWs, but the coverage is poor as evidenced by a survey of 757 public secondary and tertiary hospitals across 34 states in Nigeria which showed KMC implementation in 25% of surveyed facilities [42].

At the household level, mothers from the richest household wealth quintile had higher odds of practicing SSC with their newborn immediately after birth. Women from rich households may be more educated, more likely to utilize maternal health services such as ANC, delivery in health facility [35, 43, 44] and ultimately be more aware of SSC and its benefits, from their encounter with health service providers. Community level media exposure was a significant cluster characteristic affecting SSC practice between mother and newborn at birth. Lower odds of SSC was observed among mothers from communities with high rate of non-use of media. The media plays an important role in raising awareness and creating demand for maternal health service utilization and influence health seeking behaviour [45–47]; usage of these services could sensitize mothers about SSC and its benefits, and provide a platform for practice.

### Strength and limitation

This study utilized the most recent NDHS 2018, a nationally representative population-based survey. The findings contributes to knowledge, given the paucity of studies on the subject area in Nigeria. Also the application of multilevel analysis addressed the hierarchical nature of NDHS data. However, the study does have some limitations. First, the cross-sectional nature of the data precludes conclusions about causality of the factors on the SSC cannot be made. Secondly, the construction of community variables from aggregation of individual level data means that inferences at a higher level are made based on individual level data [48]. Thirdly, there may be recall bias as the data was collected retrospectively.

## Conclusion

The coverage of SSC in Nigeria is poor, despite evidence of its numerous benefits to the mother and the newborn. Moreover, this study revealed that the practice of SSC immediately after delivery between mothers and their newborn is associated with individual level factors such as place of delivery, mode of delivery, maternal educational level, birth weight of new born, ANC attendance during pregnancy; household wealth and community exposure to media.

## Data Availability

Data for this study were sourced and available here: http://dhsprogram.com/data/available-datasets.cfm.

## Abbreviations

AIC: Akaike's Information Criterion
ANC: Antenatal Care
AOR: Adjusted Odds Ratio
BIC: Bayesian Information Criterion
CS: Caesarean Section
ICC: Intra-class correlation
IMNCH: Integrated Maternal, Newborn and Child Health
KMC: Kangaroo Mother Care
LBW: Low Birth Weight
MOR: Median Odds Ratio
NDHS: Nigeria Demographic and Health Survey
SDGs: Sustainable Development Goals
SE: Standard Error
SSC: Mother and newborn skin-to-skin contact
PCV: Proportional Change in Variance
VPC: Variance Partition Coefficient
WHO: World Health Organization
